# Constrictive-effusive pericarditis as presenting feature of IgG4-related disease (IgG4-RD): a case report

**DOI:** 10.1186/s13256-026-06239-w

**Published:** 2026-06-21

**Authors:** Robert Fekete

**Affiliations:** New York Medical College, 19 Bradhurst Ave Suite 3850S, Hawthorne, NY 10532 USA

**Keywords:** IgG4-RD, Constrictive-effusive pericarditis, Rilonacept, Rituximab, Case report

## Abstract

**Background:**

IgG4-Related Disease (IgG4-RD) is a fibroinflammatory disorder initially identified as autoimmune pancreatitis but subsequently shown in numerous other organs with common pathological hallmarks. Constrictive pericarditis is an unusual presentation of IgG4-RD.

**Case report:**

This case report is about a 45-year-old previously healthy Slavic male patient with complicated course of IgG4-Related Disease (IgG4-RD) with pancreatic cyst, constrictive-effusive pericarditis potentially triggered by 3-month antecedent Coxsackie virus infection with hand foot and mouth syndrome, who had 495 cc pericardial fluid drained, bilateral pleural effusions with 1 L fluid drained from the right side. He was treated with methylprednisolone oral up to 48 mg daily. With each decrease in steroid dose (given maximum glucose on metabolic panel to 903) or holiday in steroid up to 6 weeks, he experienced worsening of positional and exertional shortness of breath. Final diagnosis and resolution of symptoms were obtained after pericardiectomy showing abundant fibrosis and plasma cells staining for IgG4. Remission was maintained on rituximab infusion.

**Conclusion:**

This case highlights difficulty in diagnosing and treating IgG4-RD. His initial IgG4 serum level was normal to only mildly elevated. Definitive diagnosis was via tissue biopsy which required open heart surgery to access. Prior to the diagnosis, he was treated with methylprednisolone which led to severely elevated glucose levels. Oral steroids could not stop the progression of constrictive pericarditis. Pericardiectomy and subsequent treatment with rituximab led to continued remission.

## Background

IgG4-Related Disease (IgG4-RD) is a fibroinflammatory disorder initially identified as autoimmune pancreatitis but subsequently shown in numerous other organs with common pathological hallmarks [1, 2]. Constrictive pericarditis is an unusual presentation of IgG4-RD.

## Case report

This case report is about a 45-year-old previously healthy Slavic male patient with complicated course of IgG4-Related Disease (IgG4-RD) with pancreatic cyst and constrictive-effusive pericarditis. One hypothesis for triggering the pericarditis is a 3-month antecedent Coxsackie virus infection with hand foot and mouth syndrome. Serum testing was positive for Coxsackie A7, A9, A16, A24 IgG at 1:1600. Patient began to develop progressive ascites, increased abdominal girth, as well as progressive bilateral lower extremity edema (Table 1).

**Table 1:** Timeline of key clinical events

Time	Clinical event
3 months prior	Hand foot and mouth syndrome, development of ascites and bilateral lower extremity edema
Initial admission	Drainage of pericardial and pleural effusions, initiation of colchicine and indomethacin
1 month	Switch to methylprednisolone oral 48 mg daily
4 months	Treatment of steroid-induced diabetic ketoacidosis with intravenous insulin, lowering methylprednisolone oral to 2 mg twice daily
6 months	Initiation of rilonacept in addition to methylprednisolone 2 mg twice daily
11 months	Pericardiectomy and cessation of steroid and rilonacept
13 months	Serum IgG4 level elevated to 162.5 mg/dL
14 months	Initiation of rituximab IV infusion

He presented to the emergency room with exertional and positional shortness of breath. He had nighttime orthopnea. Physical examination was significant for anasarca, jugular venous distention, Kussmaul sign, tachycardia, enlarged and pulsatile liver, and normal blood pressure. Electrocardiogram had decreased QRS amplitude but without electrical alternans. Emergent echocardiogram showed large pericardial effusion and signs of constriction including septal bounce.

**Fig. 1:**
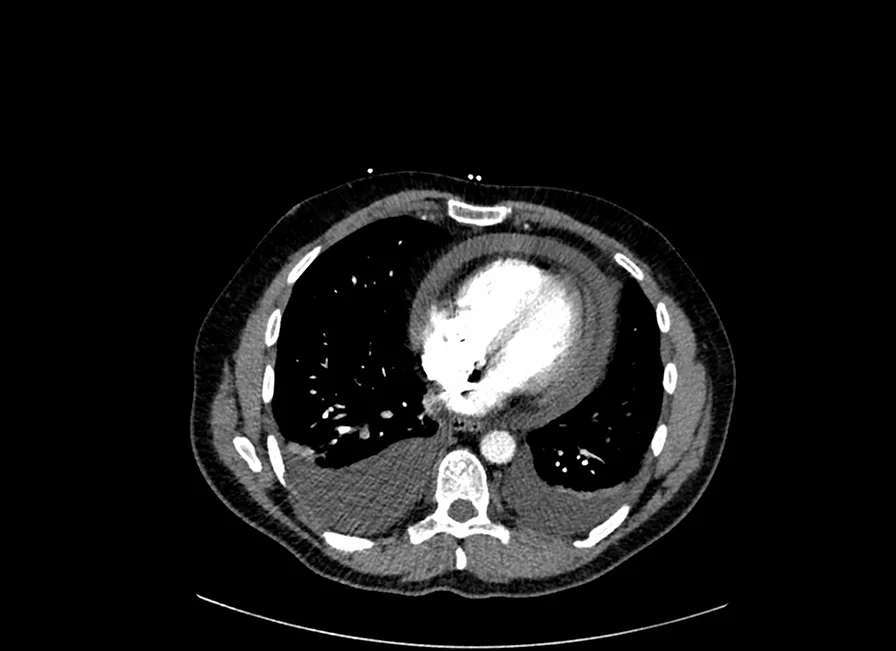
CT chest showing large bilateral pleural effusions as well as pericardial effusion and thickened pericardium

He had 495 cc pericardial fluid drained, which was sanguinous. There were also bilateral pleural effusions (Fig. 1). Pleurocentesis was performed with 920 cc of yellow serous fluid which was transudative by Light’s criteria:

lactate dehydrogenase fluid 74, serum 234 (units per liter), total protein fluid 1.9, serum 6.5, (grams per deciliter), albumin fluid 1.3, and serum 3.3 (grams per deciliter). CD38-positive T cells and B cells were demonstrated on cytology.

Adenosine deaminase in pleural fluid was within normal at 2.4 U/L (reference range < 9.2 U/L). Initial IgG4 levels, C3, C4, anti-nuclear antibody, anti-double stranded DNA antibody, and serum interferon-gamma release assay (IGRA) for tuberculosis were negative. C-reactive protein level during the initial admission was 11 mg/dL (normal < 0.5 mg/dL). Bone marrow aspiration, X ray skeletal survey, and whole-body positron emission tomography were normal. He was discharged from the hospital and placed on indomethacin 50 mg BID and colchicine 0.6 mg BID. Given the return of exertional shortness of breath on this treatment for one month, he was switched to methylprednisolone oral up to 48 mg daily.

Unfortunately, he developed excessive thirst, frequent urination, and tiredness on this steroid dose. Serum glucose was elevated to 903. He was admitted to the medical intensive care unit with glucose of 423 with ketoacidosis and ketonuria. Serum acetone was present. Serum beta hydroxybutyrate level was 6.56 mmol/L (normal 0.02–0.27 mmol/L). Urinary ketones were > 150 mg/dL. He was treated with intravenous insulin. Blood glucose subsequently stabilized on methylprednisolone 2 mg twice a day and weekly rilonacept 220 mg subcutaneous treatment. Rilonacept is a soluble receptor which traps interleukin-1 [3]. Rilonacept allowed for reduction of methylprednisolone, but did not reduce constriction. This may be due to the patient’s already advanced fibrotic constrictive pericarditis.

**Fig. 2:**
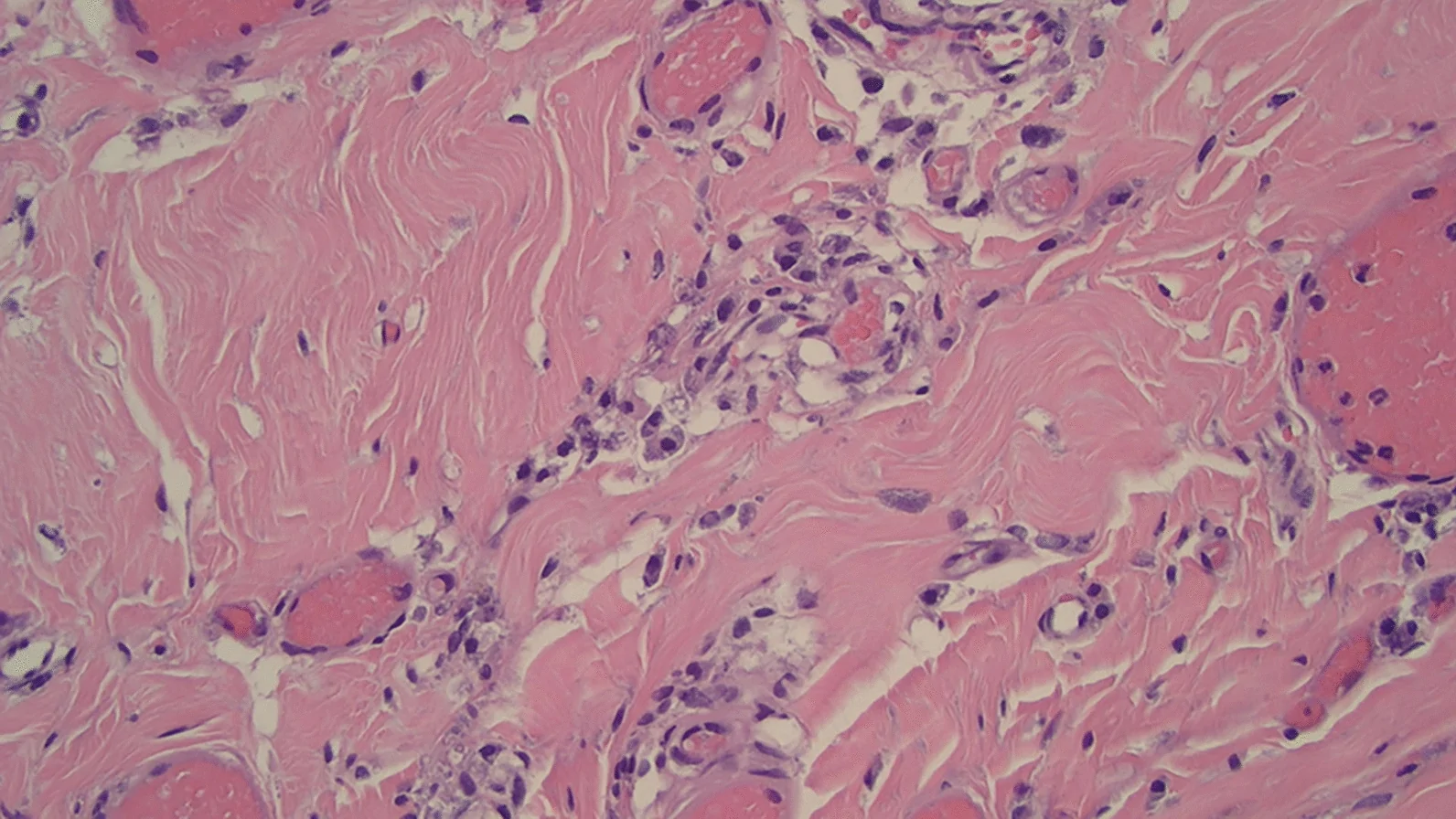
Hematoxylin—eosin stain showing abundant plasma cells and fibrosis of pericardium, 100X magnification

**Fig. 3:**
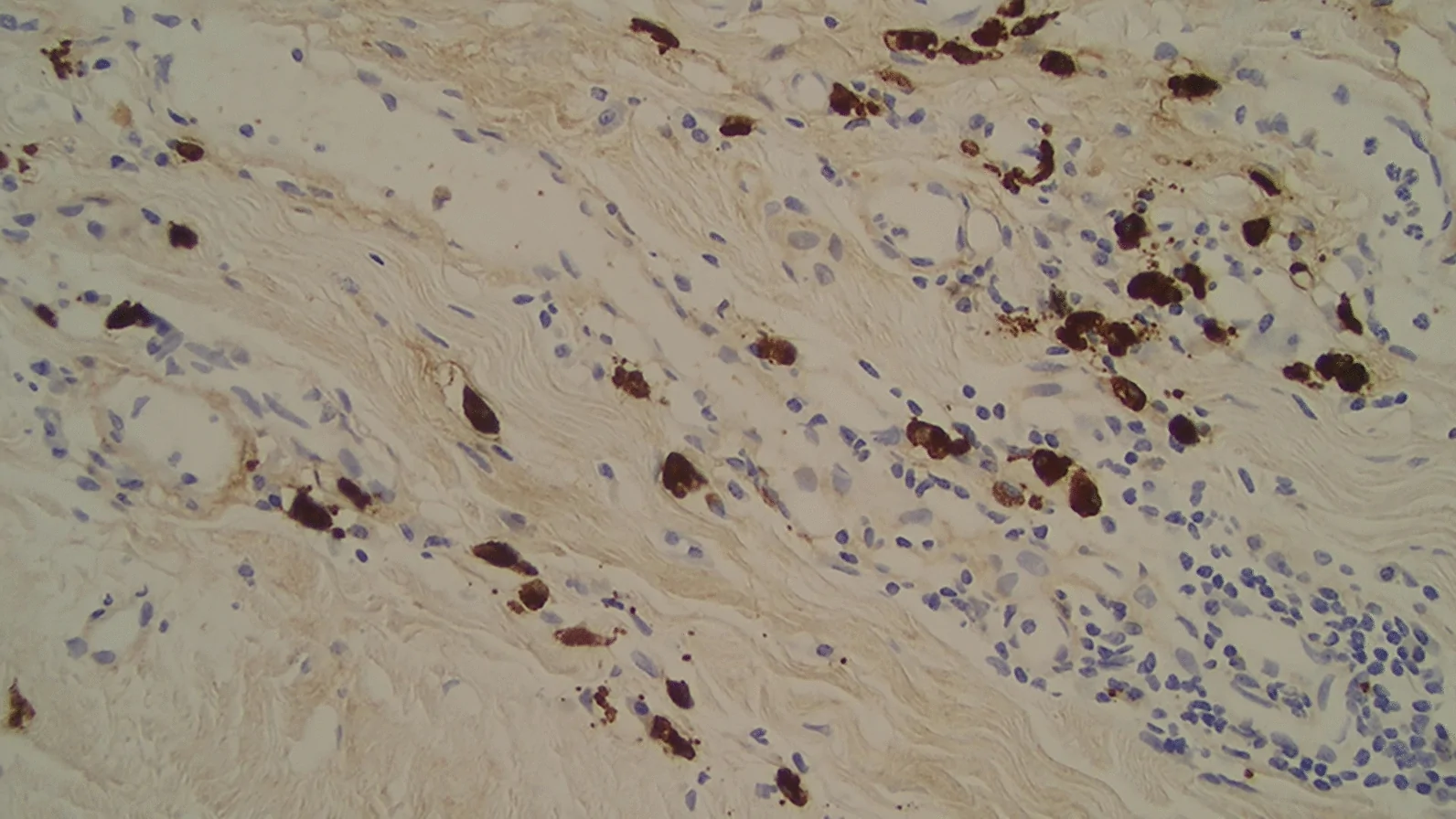
IgG4 plasma cell immunostaining of pericardium, 100X magnification

**Fig. 4:**
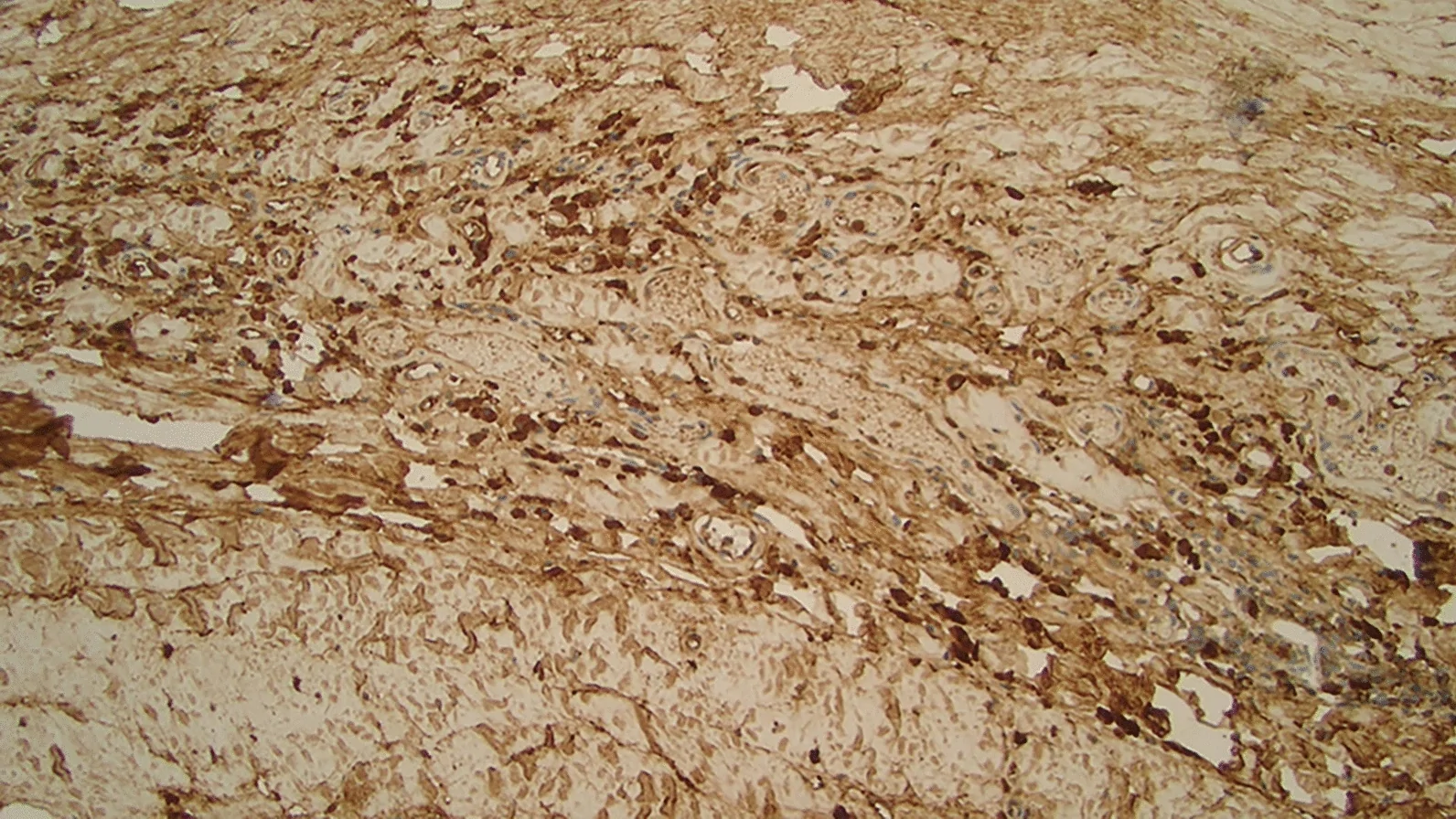
IgG total immunostaining of pericardium, 40X magnification

Diagnosis of IgG4-RD was made via pericardial biopsy during curative pericardiectomy 11 months after emergency room presentation. It showed storiform fibrosis and abundant lymphocyte and plasma cell infiltration (Fig. 2) with more than 50% of IgG plasma cells staining for IgG4 (Figs. 3, 4). Obliterative phlebitis was not present. There were more than 60 IgG4-positive plasma cells per high power field. IgG4 level was normal to mildly elevated during initial eval which is a recognized feature of this disease. IgG4 level increased to 162.5 mg/dL after complete cessation of steroid. At this time, the patient met the 2020 revised comprehensive diagnostic (RCD) criteria as definite IgG4-RD [1] given organ involvement, IgG4 serum level above 135 mg/dL, dense lymphocyte and plasma cell infiltration with fibrosis, ratio of IgG4-positive plasma cells / IgG-positive cells greater than 40% and the number of IgG4-positive plasma cells greater than 10 per high powered field, and storiform fibrosis.

Remission was maintained with intravenous rituximab treatment of 1000 mg weekly for two doses every six months. Favorable outcomes are achievable with appropriate therapy.

The patient likely had additional congestive hepatopathy or steroid-induced metabolic changes with elevated Gamma-Glutamyl Transferase (GGT) to 684 IU/L (normal 0–65 IU/L) and alkaline phosphatase to 235 IU/L (normal 44–121 IU/L) which resolved after pericardiectomy. There was also significant proteinuria with improvement on steroids, worsening after steroid holidays, and complete resolution after pericardiectomy. Minimal change disease from the initial Coxsackie infection is a potential hypothesis for this finding. The highest random total protein to creatinine ratio was 6058 mg protein/gram creatinine. Renal biopsy showed mild chronic changes with about 9% global glomerulosclerosis and mild interstitial fibrosis and tubular atrophy.

Experimental biomarkers of IgG-RD were present before pericardial biopsy was obtained. Chemokine (C-X-C Motif) Ligand 9, (CXCL9), at 1180 pg/ml, normal < 647 pg/mL, shown to be a putative biomarker of IgG4-RD and follicular lymphoma [2]. There was also elevation of cytokines. Interleukin-6 peaked at 24.5 pg/ml (normal < 5 pg/ml) during the initial hospitalization for drainage of pericardial fluid and then resolved. About 8 months after presentation, during a six-week steroid holiday, soluble interleukin 2-receptor alpha (sIL-2Ralpha) peaked at 13,285 pg/ml (normal up to 1891 pg/ml), indicating the T cell component of the disease [4]. Interferon-gamma was elevated to 7.1 pg/ml (normal <  = 4.2 pg/ml) [5]. Transforming growth factor Beta-1 (TGF-Beta-1) which is shown to be a part of models of signaling in IgG-RD was elevated to 21,400 pg/ml (pre-11/19/2024 reference range 344–2382 pg/mL) [2, 5].

## Discussion

In the initial phase of the disease, serum IgG4 levels were normal to mildly elevated. The highest pre-surgical level was 132 mg/dl (nl 2–96 mg/dl). Profoundly elevated IgG4 levels that are a hallmark of the disease at least above the 135 mg/dl cutoff of the 2020 revised RCD for IgG4-RD were not present until after pericardiectomy and complete cessation of steroid therapy [1].

Initial differential diagnostic considerations focused on systemic lupus erythematosus (SLE), tuberculosis, and cancer-related constrictive-effusive pericarditis including multiple myeloma.

From the SLE perspective, C3, C4 anti-nuclear antibody, and anti-double stranded antibody were normal. Renal biopsy was not indicative of SLE. Acid fast bacterial cultures of pericardial fluid and surgically excised pericardium were negative for tuberculosis, in addition to negative serum IGRA testing and normal pleural fluid adenosine deaminase levels. Regarding cancer, skeletal survey and whole-body nuclear scanning were negative for masses. The patient also had a normal bone marrow biopsy and no M-protein on serum protein electrophoresis.

Pericardiectomy is the definitive treatment for constrictive pericarditis. It was first performed in 1913 by Ludwig Rehn [6]. Therapy with non-steroidal anti-inflammatory agent, indomethacin, colchicine, and oral steroid methylprednisolone was attempted first, as there may be subacute cases with evidence of ongoing inflammation who could have clinical improvement on 8–12 weeks of these therapies. These cases would be diagnosed as transient constrictive pericarditis by European Society of Cardiology guidelines [7, 8]. Rilonacept, which traps cytokines interleukin-1 alpha and interleukin-1 beta, was used given colchicine resistant pericarditis with elevated serum inflammatory markers at a time when the diagnosis was unknown [9]. The goal of therapy was to attempt to reduce constriction by reducing pericardial inflammation, but in the setting of the patient's advanced fibrotic constrictive pericarditis, anti-inflammatory biologic therapy would not be expected to reverse mechanical restriction.

Constrictive pericarditis occurs in fewer than 1% of cases of idiopathic pericarditis [8]. Other causes include rheumatological disorders including SLE, cancer, trauma, prior cardiac surgical procedures, and tuberculosis [10].

IgG4-RD is a rare disease with overall prevalence of cases in Japan estimated at 10.1 per 100,000 [11]. Pericardial involvement in IgG4-RD is considered to be extremely rare. A pediatric registry study of 13 centers reported only one case (2.9%) of pericardial involvement in this disease [12]. Pericardial involvement has been recognized as a part of multisystem IgG4-RD as opposed to an isolated or primary presentation. In this patient, it is possible that there were other unrecognized areas of organ involvement by IgG4-RD. In retrospect, the elevated serum glucose, GGT, and alkaline phosphatase may have indicated additional IgG4-RD hepatobiliary or pancreatic organ involvement, but these values could have been due to other causes such as congestive hepatopathy and steroid-induced metabolic effects. Rituximab was chosen as a steroid sparing agent which was shown to maintain remission of IgG4-RD flares when dosed every six months in an 18-month study [13].

## Conclusion

Diagnosis of IgG4-related disease in the early phase with the main lesion being in a difficult to access area such as the pericardium is very challenging. Small salivary gland biopsy after the patient was already started on steroids showed sialadenitis but not IgG4 staining. Clinicians can be alert to clues such as clinical deterioration with each steroid holiday or steroid dose reduction. Elevation of experimental biomarkers such as CXCL9, sIL-2Ralpha, and TGF-beta-1 can include IgG4-RD as a differential diagnostic consideration until definitive diagnosis can be made by pathology.

## Data Availability

All supporting data were included in this manuscript. Patient clinical data are protected by the United States Health Insurance Portability and Accountability Act (HIPAA) of 1996.
